# Encapsulation of Snail Slime in Metal–Organic Framework ZIF-8

**DOI:** 10.3390/jfb16120443

**Published:** 2025-11-28

**Authors:** Maria Cristina Cassani, Francesca Bonvicini, Maria Francesca Di Filippo, Barbara Ballarin, Silvia Panzavolta, Valentina Di Matteo

**Affiliations:** 1 Department of Industrial Chemistry “Toso Montanari”, University of Bologna, Via Piero Gobetti 85, 40129 Bologna, Italy; maria.cassani@unibo.it (M.C.C.); barbara.ballarin@unibo.it (B.B.); 2 Center for Industrial Research-Advanced Applications in Mechanical Engineering and Materials Technology (CIRI-MAM), University of Bologna, Viale del Risorgimento 2, 40136 Bologna, Italy; 3 Department of Pharmacy and Biotechnology, University of Bologna, Via Massarenti 9, 40138 Bologna, Italy; francesca.bonvicini4@unibo.it; 4 Department of Chemistry “G. Ciamician”, University of Bologna, Via Piero Gobetti 85, 40129 Bologna, Italy; mariafrancescadf@icloud.com; 5 Center for Industrial Research-Fonti Rinnovabili, Ambiente, Mare e Energia (CIRI-FRAME), University of Bologna, Viale del Risorgimento 2, 40136 Bologna, Italy; 6 Health Sciences and Technologies—Interdepartmental Center for Industrial Research (HST–ICIR), Alma Mater Studiorum—University of Bologna, Ozzano dell’Emilia, 40064 Bologna, Italy

**Keywords:** Zeolitic Imidazolate Framework-8, *Cornu aspersum* mucus, biomimetic mineralization, bactericidal activity

## Abstract

The literature consistently identifies Zeolitic Imidazolate Framework-8 (ZIF-8) as an excellent material for on-demand drug delivery. Its appeal results from its superior loading capacity, inherent stability within physiological environments, and the ability to fine-tune its drug release kinetics. In this work, we investigated the encapsulation of snail slime extracted from *Cornu aspersum* mucus into ZIF-8. PXRD, SEM microscopy, ATR-FTIR spectroscopy, and fluorescence microscopy were used for a detailed characterization of the nanoparticles. The antibacterial potential of the ZIF-8-based biocomposite was assayed in vitro against *Staphylococcus epidermidis*. Overall, the results indicate that encapsulating the snail slime within ZIF-8 enhances its antibacterial activity, yielding a potent antimicrobial material.

## 1. Introduction

Zeolitic Imidazolate Framework-8 (ZIF-8), constructed from 2-methylimidazolate and zinc ions, is a subclass of MOFs that is chemically stable in aqueous and basic media and has a surface area of up to ca. 1800 m^2^g^−1^ [1]. These physical properties, the nano-sized pore dimensions of ZIF-8 (ca. 11.6 Å) [2], and its pH-responsive dissolution behavior in acidic solutions have motivated the investigation of ZIF-8 for biomedical applications.

Our research group already studied the use of ZIF-8 as coating to synthesize composite materials useful in wound dressing and to prevent implant-associated infections [3,4]. However, in the literature, this material is also studied as drug delivery system for cancer therapy [5,6,7,8,9]. It has been shown that ZIFs can be used for the encapsulation of a large variety of systems ranging from small molecules to biomacromolecules (proteins, enzymes, horse-radish peroxidase HRP, bovine serum albumin BSA, deoxyribonucleic acid DNA, insulin, and glycosaminoglycans GAGs) [5,6,7,8,9,10,11,12,13,14,15,16,17,18,19,20,21] to form bioactive composites, via a one-pot synthetic approach termed “biomimetic mineralization”, due to its similarity to natural biomineralization processes, in which proteins spontaneously induce the formation of minerals. The creation of protein@MOF biocomposites occurs when negatively charged biomacromolecules seed the rapid, surface-localized growth of an MOF shell. A significant advantage of this MOF coating is its dual function: it protects the enclosed enzyme from hostile environments (such as high temperatures or breakdown by proteases) and simultaneously employs its porous structure to ensure size-selective access for substrates to the active site.

In this work, taking advantage of its negative ζ potential (28.5 mV), we applied the biomimetic mineralization synthesis for the encapsulation of snail slime extracted from *Cornu aspersum* snails [22]. Snails belong to the phylum Mollusca, the second-largest phylum in the animal kingdom, encompassing at least 80,000 species. While most mollusks are aquatic, this group is found globally, including some terrestrial species. All commercially farmed snails are land snails belonging to the class Gastropoda. In Europe, among the wild species, only about a dozen are considered edible, with just four or five being widely commercialized. Notably, *Cornu aspersum* dominates this market, accounting for 40% of sales.

Almost all of the extraction methods consist of collecting snail slime, which is then settled, filtered, concentrated, and dried according to different procedures, either at low temperatures under vacuum evaporation, or by freeze-drying or spray-drying. The outcoming aqueous solutions can be stored under aseptic conditions within sterile vials after filtration with specific filters or by the addition of antiseptic substances such as glycerin. Solutions can then be dialyzed to eliminate all the chemicals added by the suppliers like preservatives and stimulating solutions, as well as some chlorides that can be present in the slime. The mucus of *Cornu aspersum* has been valued since ancient times for treating human disorders, especially skin conditions, through the direct application of the raw secretion. In fact, snail mucus is a mixture of active compounds such as proteins, glycoproteins, fatty acids, polyphenols, vitamins, glycolic acid, allantoin, minerals, carbohydrates, and glycosaminoglycans. The latter have a role in the regulation of physiological processes through interactions with several proteins and accounting for the hygroscopic activity of snails to avoid dehydration. More recently, its use has expanded globally as an ingredient in cosmetic products. Furthermore, it is suggested for use in parapharmaceutical formulations to manage wounds and treat chronic bronchitis. Despite this widespread commercial success, the chemical composition, specific biochemical characteristics, and precise biological effects of *Cornu aspersum* mucus are currently supported by limited scientific data, especially in composite materials [22,23].

Further uses of snail slime are related to its physical characteristics; in fact, this substance acts as both glue and a lubricant. Moderate anti-inflammatory effect on acute edema and considerable antioxidant activity, owing to free radical scavengers such as flavonoids and phenols, are other virtues ascribed to snail slime. Other studies also confirmed the antibiotic activity of snail slime. Recently, snail slime has also been used to induce the formation of gold and silver nanoparticles [22,24]. This study reports the synthesis and characterization of a biocomposite based on ZIF-8 incorporating a selected snail mucus that was previously characterized [23]. The encapsulation of snail slime into ZIF-8 is worth investigating due to the well-documented biological properties of snail secretion, which has shown promising applications in drug delivery systems [25,26] and in the formulation of chitosan-based biofilms. Snail slime is rich in bioactive compounds such as glycoproteins and enzymes that promote cell regeneration, reduce inflammation, and exhibit antimicrobial effects. The obtained biocomposites could be integrated into various delivery platforms, including polymeric patches, hydrogels, composites, and treated bandages, for potential applications in cutaneous wound healing and bone tissue infection treatment.

## 2. Materials and Methods

**Materials.** Chemicals and solvents were utilized as received from Sigma-Aldrich (now Merck KGaA, Darmstadt, Germany). All experimental procedures relied on ultrapure water (>18 MΩ cm) purified via the Milli-Q plus system (Millipore Co, Burlington, VT, USA). The snail slime obtained from *Cornu aspersum* snails was kindly donated by ‘lumacamadonita’ (“madonita snail” in English, www.lumacamadonita.it, accessed on 1 may 2023, Palermo, Italy), and for this reason, hereafter, we use the abbreviation MAD to indicate the snail mucus. A commercial snail slime solution, prepared by diluting extracted slime with purified water, was used in these studies. The snails, raised outdoors on land and fed fresh vegetables, were harvested three times annually. After washing, they underwent ozone treatment for disinfection before the slime was collected via mechanical stimulation using the patented EXTRACTA machine—a cruelty-free method yielding slime with a pH between 5 and 7.

Prior to experimental use, the slime was further purified via dialysis. This involved placing a known volume of slime into a cellulose dialysis tubing membrane (Merk, 12–14 kDa cutoff) that had been pre-washed with distilled water to remove the retailer’s humectant. The membrane was then submerged in distilled water and stirred for 24 h, with the water being replaced twice to ensure the complete removal of additives. The resulting dialyzed samples (pH ≈ 7, ζ potential ≈ −28.5 mV) were either stored at −19 °C or lyophilized after freezing and stored between 0 °C and 4 °C. Characterization results for both the as-received and dialyzed slime have been previously reported [22,23].

**Fluorescent Labeling of MAD.** First fluorescein isothiocyanate isomer I was dissolved at a concentration of 1.0 mg/mL of dimethyl sulfoxide (DMSO); successively, 120 μL of such solutions are added to a 10 mg/mL solution of lyophilized MAD in carbonate-bicarbonate aqueous buffer solution (0.1 M, pH 9.2) and left for 2 h in darkness under gentle stirring. The fluorescein-tagged snail slime (F-MAD) was recovered by eluting the reaction mixture through a Sephadex G-15 column (Sigma-Aldrich) using a phosphate-buffered saline solution (PBS, pH 7.4). Thereafter, the eluted fluorophore-tagged MAD solution was passed again through a Sephadex G-15 column to ensure the complete removal of unreacted fluorophore molecules. The crude solution was then concentrated through a 10 kDa membrane (Amicon^®^ Ultra-15 Centrifugal Filter Unit, Millipore) by centrifugation (6000 rpm for 20 min), followed by solvent exchange with ultrapure water (6000 rpm for 20 min). The concentration/solvent-exchange procedure was performed twice to ensure the complete removal of buffer salts. The resulting fluorophore-tagged MAD solution was then lyophilized and stored in the dark at 4 °C (Figure S1).

**Synthesis of ZIF-8.** The synthesis of ZIF-8 was carried out mainly following a literature method [14,27]: 5 mL of an aqueous solution of Zn(OAc)_2_·2H_2_O (0.4 mmol, 80 mM) was rapidly added to a solution of 2-methylimidazole (5 mL, 6.4 mmol, 1.28 M). The synthesis mixture (molar ratio Zn:2-HmIM:H_2_O = 1:16:1389) was stirred at 400 rpm for 24 h at room temperature. The resulting suspension was then centrifuged and washed repeatedly with CO_2_-free water until the wash liquid reached a pH of approximately 7 (as confirmed by litmus paper). The material was subsequently air-dried in an oven at 43 °C for another 24 h. For thermal activation, the white powder was treated at 100 °C under a vacuum of 10^−2^ mbar. This yielded 79 mg of the activated product (yield: 87% based on zinc; Figure S2.1). Elemental analysis confirmed the zinc content: 27.8 ± 0.4% (theoretical for Zn(mIM)_2_: 28.7%). The activated material was stored in a desiccator.

**Synthesis of MAD@ZIF-8.** In a typical experiment, 2 mL aqueous solution of Zn(OAc)_2_·2H_2_O (80 mM) was rapidly poured into a 2 mL aqueous solution containing 2-HmIM (1.28 M) and lyophilized MAD at different concentrations (from 2 mg/mL to 5 mg/mL in the premix solution) at room temperature (Zn:2-HmIM:H_2_O = 1:16:1375). The resulting suspension was stirred at 400 rpm for 24 h at room temperature. Subsequently, the material was centrifuged and washed repeatedly with CO_2_-free water until the washing reached a neutral pH (checked by litmus paper). The collected solid was then dried in an oven at 40 °C for 24 h and finally stored at 4 °C. Depending on the amount of MAD employed, the final powder presents a whitish to creamy color (Figure S2). The fluorophore-tagged F-MAD@ZIF-8 was prepared with the same procedure with the exception that the concentration of the fluorophore-tagged MAD was 1.0 mg/mL (premix solution) in all cases.

**Synthesis of F-MAD-*ON*-ZIF-8.** The fluorophore-tagged F-MAD-*ON*-ZIF-8 was prepared by adding, to a solution of previously synthesized ZIF-8, 1.0 mg/mL of F-MAD, typically in a F-MAD:ZIF-8 ratio of 1:16. The mixture was stirred for 1 h, then centrifuged and washed with CO_2_-free water until the washings had a neutral pH (litmus paper), followed by drying in an oven at 40 °C for 24 h, and finally stored at 4 °C.

**Characterization.** ATR-FTIR analyses were performed with a Perkin Elmer Spectrum Two spectrophotometer (Waltham, MA, USA), equipped with a Universal ATR accessory, in the range of 4000–400 cm^−1^ and a resolution of 0.5 cm^−1^. Samples were directly analyzed after being ground in a mortar, performing 40 scans for each analysis. Powder X-Ray Diffraction (PXRD) patterns were recorded in reflection mode by using a Philips X’Celerator equipped with a graphite monochromator. The 2θ range was from 4 to 40°, with a step size of 0.100° and time per step of 120 s. CuKα (40 mA, 40 kV, 1.54 Å) was used. Morphological investigation was performed by Scanning Electron Microscope (SEM, Oxford Instruments, Abingdon, United Kingdom)), and images were obtained using a Leica/Cambridge Stereoscan 360 with INCA software. “Digimizer” software (version 5.8.0, MedCalc Software Ltd., Ostend, Belgium) was used to estimate the mean dimensions, based on at least 100 data points per sample. Thermogravimetric analysis (TGA) was performed by Q600 SDT-TA instruments (New Castle, DE, USA). The analysis was performed under a nitrogen flow (100 mL/min), at a rate of 3.5 °C/min up to 120 °C and then 10 °C/min to 800 °C. The overall amount of zinc present in the different samples was determined by means of Agilent 4210 Molecular Plasma-Atomic Emission Spectroscopy (MP-AES, AgilentSanta Clara, CA, USA. Zinc lines at 472.215 and 481.053 nm were used. The analyses were conducted by comparison with five calibration standards (2, 20, 30, 50, 100 ppm), prepared by dilution to 100 mL of a 1000 ppm zinc standard (Carlo Erba Reagents). The samples were analyzed after treating the solids (ca. 10 mg) with 1.0 M nitric acid in 50 mL; results from this analysis represent the mean value of three different determinations. The adsorption isotherm was measured by using a static volumetric apparatus (ASAP 2020 Surface Area and Pore Size Analyzer, Micromeritics, Norcross, GA, USA). The samples were degassed at 10^−3^ mbar at 100 °C (ramp rate of 10 °C/min) for 2 h prior to measurement; MicroActive Software (version 4, Micromeritics Instrument Corporation, Norcross, GA, USA)was used for data elaboration. Langmuir and Brunauer–Emmett–Teller (BET) isotherms models were used for the estimation of the specific surface area (S_bet_ = BET surface area and S_L_ = Langmuir surface area) according the reference [28].

The presence and spatial distribution of the fluorophore-tagged biomolecules in (or on) the MOF composites were determined using fluorescence microscopy (Nikon Eclipse E400) equipped with a 100 W mercury lamp (C-SHG1, Nikon Corp., Japan) and a 450–490 nm filter. Spectrofluorimetric analyses were performed on the fluorescent-tagged samples with a spectrofluorometer by Edinburgh Instruments, using an excitation wavelength of 492 nm and emission’s wavelength of 520 nm; the lamp was Xe900. The calibration curves are performed using F-MAD at different concentrations (Figure S3) in different buffers in order to study the different encapsulation efficiencies.

**Kinetic studies.** Following literature procedures [29], we applied two different kinetic models to our release study data and evaluated the best fitting curve. The first model applied was the Higuchi model, the first mathematical model for determining the release mechanism of drugs from matrix systems proposed by Higuchi in 1963. This model was applied to study the release of water-soluble or poorly soluble drugs incorporated into semi-solid and solid matrices. The model is expressed by the following mathematical equation:

Q = A[D (2C − Cs) ⋅ Cs ⋅ t]^1/2^ or Q = Kt^1/2^

Here, Q represents the amount of drug released from a unit surface, A, at time t, in mg. C is the initial concentration of the drug, in mg/mL, Cs is the concentration of the drug dissolved in the medium, and D is the diffusion coefficient (which characterizes the diffusion of drug molecules in the matrix). The simplified Higuchi model expresses the release of drugs from insoluble matrices as a dependence on the square root of time based on the Fickian diffusion equation.

The second model used was the Korsmeyer–Peppas model. Korsmeyer and others proposed a simple equation describing the release of drug substances from polymer systems. Korsmeyer and Peppas developed an empirical equation to analyze both Fickian and non-Fickian release from swellable and non-swellable gels and materials. To determine the mechanism of drug release, the data for the first 60% of the release were checked using the Korsmeyer–Peppas model. The equation is mathematically expressed as follows:

Mt/Mα = Ktn

Here, Mt/Mα is the fraction of the drug released at time t. K is the release constant, which reflects the structure and geometric characteristics of the delivery system. n is the release exponent, which indicates the drug’s delivery mechanism through the polymer. The value of n can be used to characterize different release mechanisms.

**Antibacterial studies.** The antibacterial properties of the MAD@ZIF-8 sample were assessed in vitro against the reference laboratory strain of *Staphylococcus epidermidis* ATCC 12228, obtained from the American Type Culture Collection (ATCC, Manassas, VA, USA). The bacterial colonies were routinely cultured on 5% sheep blood agar plates (Biolife Italiana s.r.l., Milan, Italy) at 37 °C for 18–24 h. For antimicrobial testing, the powders, MAD@ZIF-8, ZIF-8 and MAD, were resuspended in Mueller–Hinton broth (MHB, Biolife Italiana S.r.l., Milan, Italy) at a final concentration of 10 mg/mL. Suspensions were freshly prepared prior to experiments and maintained under vigorous agitation to ensure uniform dispersion. Serial two-fold dilutions were prepared in MHB in the range of 1–0.03 mg/mL; thereafter, the bacterial inoculum of *S. epidermidis* was added at 10^8^ CFU (colony-forming units)/mL. After 24 h of incubation at 37 °C, bacterial growth was assessed by measuring the optical density at 600 nm using a spectrophotometer. In addition, aliquots of the bacterial cultures were serially diluted and plated on Plate Count Agar (PCA, Biolife Italiana S.r.l., Milan, Italy) to determine viable cell counts, allowing colonies to form from living bacteria. Plates were incubated at 37 °C for 24 h, after which CFUs were imaged (iBright 1500 Imaging Systems, Thermo Scientific).

**Statistical analysis.** The antibacterial activity of the samples was expressed in terms of MIC (Minimum Inhibitory Concentration) for *S. epidermidis*. Growth percentage values of the reference strain were determined relative to the untreated positive controls (bacterial cells incubated in MHB). The lowest concentration of samples that inhibited bacterial growth (≥90%) was noted as the MIC value. All assays were carried out in triplicate and in at least two independent assays. One-way analysis of variance (one-way ANOVA), followed by Dunnett’s multiple comparisons test, was used to compare the antibacterial activity of the sample at the different concentration.

## 3. Results and Discussion

### 3.1. Characterization of As-Synthetized ZIF-8 Crystals

For a comparison with the biomimetically mineralized materials, ZIF-8 was synthesized by rapidly adding an aqueous solution of Zn(OAc)_2_·2H_2_O (80 mM) to a solution of 2-methylimidazole (1.28 M) using a stoichiometric ratio HmIM/Zn of 16:1 and stirring for 24 h at room temperature. The chosen parameters reflect the requirements for aqueous ZIF-8 synthesis reported in the literature: efficient formation of highly crystalline sodalite-type ZIF-8 demands high concentrations and a large excess of the organic ligand, which contrasts with syntheses in organic solvents [27,30,31,32].

**Figure 1:**
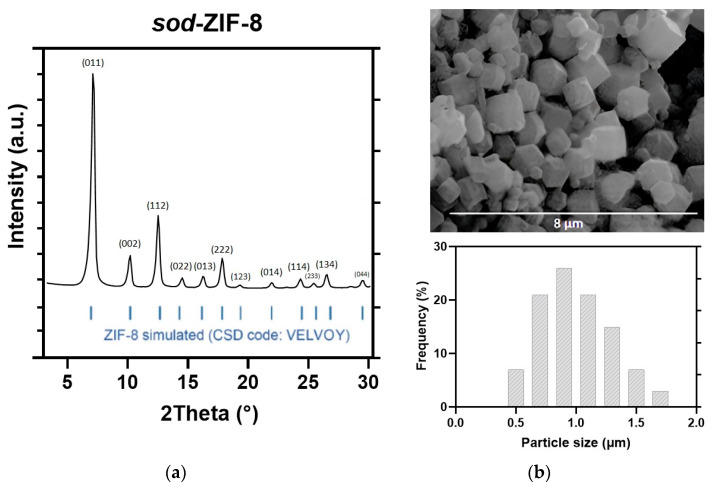
(**a**) XRD pattern, (**b**) SEM image, and particle size distribution of the as-synthesized ZIF-8.

The crystal structure and morphology of the *activated* ZIF-8 product have been examined by PXRD and SEM. The results are illustrated in Figure 1 and are consistent with several previous studies. The XRD analysis (Figure 1a) confirms the presence of a single-phase sodalite topology, with the characteristic reflections at 2θ = 7.30° (011), 10.35° (002), 12.70° (112), and 18.00° (222). The SEM analysis (Figure 1b) showed the characteristic rhombic dodecahedron morphology of sod-ZIF-8 topology, with a uniform size distribution and average dimensions of 0.7 ± 0.2 µm.

**Figure 2:**
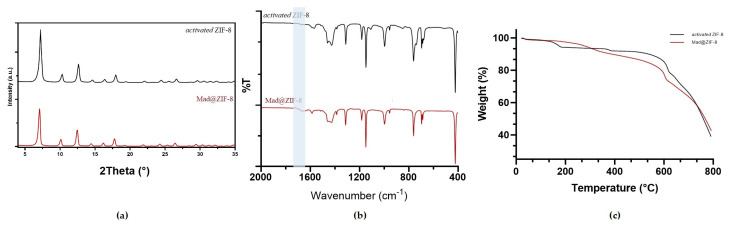
(**a**) XRD pattern, (**b**) ATR-FTIR, and (**c**) TGA of ZIF-8 (black) vs. activated Mad@ZIF-8 (red).

Figure 2b shows the ATR-FTIR spectra of the *activated* ZIF-8. All bands are consistent with those previously reported in the literature; for example, the peak at 1584 cm^−1^ is attributed to the C=N stretching mode; the bands in the spectral region of 600–1500 cm^−1^ are associated with the entire stretching or bending of the ring, while the band at 421 cm^−1^ is ascribed to the Zn-N stretching vibration [33]. The TGA pattern for the *activated* ZIF-8 sample measured under nitrogen is shown in Figure 2c (see Figure S4.1 for a comparison with the *as-synthesized* material). The initial weight loss of about 10% (from ambient temperature to ca. 200 °C) corresponds to the gradual release of water and unreacted 2-HmIM adsorbed on the surface and within the ZIF-8 framework. At higher temperatures (about 600 °C), decomposition occurs, leading to the collapse of the MOF structure, with a total weight loss of about 60%, confirming the high thermal stability of prepared ZIF [34].

N_2_ adsorption–desorption isotherm of the *activated* ZIF-8 after degassing the sample at 100 °C for 2 h at 10^−3^ mbar is shown in Figure S5a, and it indicates a surface value of 1364 (S_BET_) and 1360 (S_L_) m^2^ g^−1^ and micropore volume 0.462 cm^3^/g, values very close to those already reported in the literature for the synthesis carried out in water [20,30].

### 3.2. Synthesis of MAD@ZIF-8 Biocomposite

Previous studies have enabled the characterization of the slime employed; specifically, the slime utilized in this study was derived from a commercial solution following dialysis and lyophilization, processes which allowed obtaining a material with a protein content corresponding to 97% by weight [22,23].

The procedure for the preparation of the MAD@ZIF-8 biocomposite was identical to the above-mentioned synthesis of ZIF-8, with the exception that lyophilized MAD was first dissolved in the solution of 2-methylimidazole (1.28 M) before the addition of the zinc acetate solution [20]. Although the premix concentration of MAD was varied in the range 2÷5 mg/mL, the characterization reported here refers only to the sample made with the higher concentration employed of 5 mg/mL of MAD.

The results of the PXRD analyses indicate that the introduction of MAD at the employed concentrations does not affect the diffraction pattern, which is in good accordance with that collected for ZIF-8. Sodalite is confirmed as the only crystalline phase (Figure 2a, PXRD diffractogram for the sample made with 5 mg/mL of MAD), but a slight shift of 0.10° at lower 2-theta values has been evaluated for the diffraction pattern collected on Mad@ZIF-8, thus corroborating the encapsulation of snail slime.

The ATR-FTIR analysis (Figure 2b, sample prepared with 5 mg/mL MAD) of the biocomposites reveals, alongside the characteristic vibration bands of the ZIF framework, the presence of a weak band at around 1653 cm^−1^, typical of the amide bond, thus indicating the presence of snail mucus [22,31,35].

TGA analyses of the sample (Figure 2c, for the sample made with 5 mg/mL of MAD, Figure S4.2 TGA of the activated sample) revealed slower weight losses than those observed for the samples of ZIF-8. This could be due to an increase in thermal resistance conferred by the presence of MAD in the ZIF-8 system. The weight loss at 600 °C, attributed to the degradation of ZIF-8 (Figure 2c and Figure S4.1 and S4.2) remains consistent for both samples. Additionally, around 200 °C, corresponding to the degradation of proteins in the snail slime, the observed weight loss is minimal. This indicates that ZIF-8 provides a protective barrier, enabling the MAD to preserve its composition over a broader temperature range compared to MAD alone (Figure S4.3). Further morphology and porous structure of MAD@ZIF-8 were found by SEM and nitrogen adsorption–desorption, respectively.

**Figure 3:**
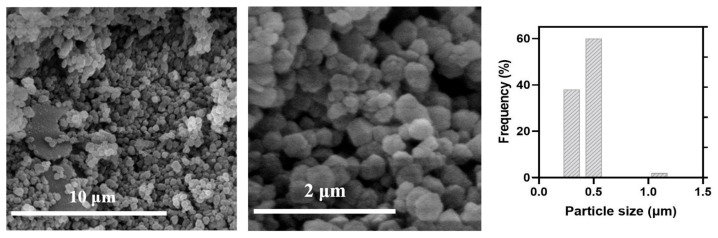
SEM images and size distribution of Mad@ZIF-8.

As observed in the image of MAD@ZIF-8 biocomposite (Figure 3), although the morphology remains the same of bare ZIF-8, the crystals are smaller, with average size of 0.4 ± 0.2 μm, which is comparable to values reported in the literature for other ZIF-8 encapsulations. The different sizes between ZIF-8 and MAD@ZIF-8 were ascribed to aggregative growth kinetics mediated by MAD, which allowed the formation of smaller ZIF-8 crystals.

Nitrogen porosimetry was employed to analyze the pore structure of MAD@ZIF-8. Similarly to what was performed for ZIF-8, such analyses were carried on sample degassed at 100 °C, yielding values of 950 (S_L_) and 850 (S_BET_) m^2^/g (Figure S5b). Due to instrument sensitivity, it was not possible to give an estimation of pore volume for the Mad@ZIF-8 sample.

### 3.3. Synthesis of Fluorophore-Labeled MAD@ZIF-8 Biocomposites

To ascertain the location of MAD within the ZIF-8 biocomposite, we labeled MAD with fluorescein isothiocyanate (FITC). Such choice was dictated from what was reported in literature [15,20], namely, that the fluorescein functionalization does not alter the surface chemistry of encapsulated materials. Indeed, we found that the zeta potential analysis of MAD was minimally affected by the fluorescein tag (–28.5 versus –29 mV). The fluorophore-tagged F-MAD was then employed (with a 1.0 mg/mL premix concentration) for the preparation of the biocomposites using the same procedure described above. PXRD, SEM, TGA, and ATR-FTIR analyses on the F-MAD@ZIF-8 sample show that the crystallinity and particle morphology are essentially identical (Figures S6–S9); in particular, the SEM images (Figure S9) confirmed an average size of 0.5 ± 0.2 μm.

**Figure 4:**
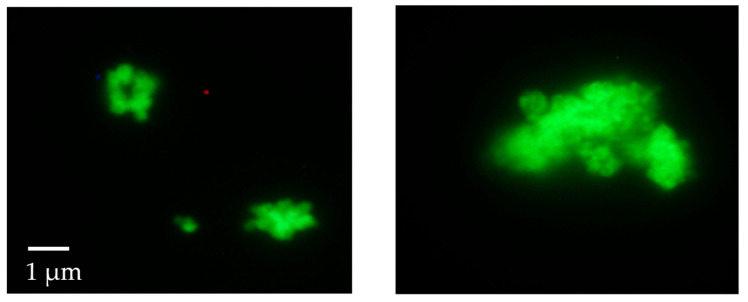
Fluorescence microscope images of the F-Mad@ZIF-8 sample before (**left**) and after (**right**) treatment with SDS (original magnification 100×).

**Figure 5:**
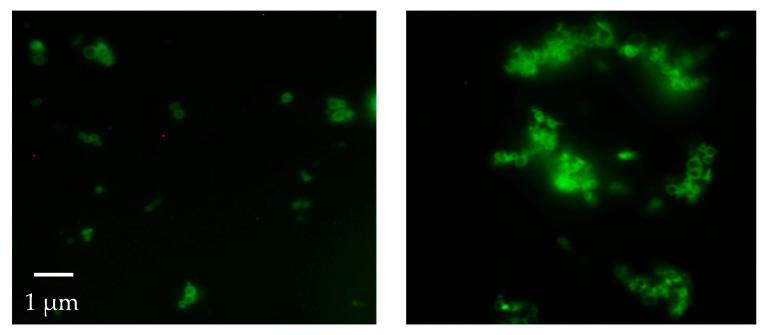
Fluorescence microscope images of the F-Mad-ON-ZIF-8 sample before (**left**) and after (**right**) treatment with SDS (original magnification 100×).

The material was then investigated using fluorescence microscopy. This analysis revealed that, contrary to what was reported by previous studies, in our case, all the attempts made (washings with water and ethanol, followed by prolonged washings with 10 wt% sodium dodecyl sulfate in TBS buffer at pH 7.5) to completely remove the F-MAD bound on the outer surface of ZIF-8 failed (Figure 4 and Figure 5).

This result was confirmed by applying the same washing protocols on samples termed F-MAD-*ON*-ZIF-8 (preparation is described in the experimental part). With regard to the encapsulated F-MAD, the images and later characterizations seem to demonstrate that it is predominantly located in the subsurface region of the sod-Zn(mIM)_2_ crystallites rather than homogeneously distributed throughout the crystal (Figure 4 and Figure 5).

To determine the immobilization efficiency of the ZIF-8, we measured the difference between the starting F-MAD used to synthesize the sample and the F-MAD present in the surfactant and washing solutions after F-MAD@ZIF-8 synthesis. Also, the work-up solutions of F-MAD-*ON*-ZIF-8 were analyzed to differentiate between the adsorbed and incapsulated MAD. We used two different pH conditions using two buffers, namely, PBS (pH 7.4) and citric buffer (pH 5), which are the two standard pH values used in the literature for this kind of analysis and also the model pHs when talking about this kind of applications.

The quantities were found as follows: After the standard synthesis of F-MAD@ZIF-8 and F-MAD-*ON*-ZIF-8, the surfactant and washing solutions were analyzed at the fluorimeter, and the concentration of remaining F-MAD was found by interpolation on the calibration curves (Figure S3). So, by comparing between the starting material and the residues in the work-up solutions, the results showed a total immobilization efficiency of the F-MAD@ZIF-8 of 98 ± 1%, corresponding to a 68% of adsorbed F-MAD and 30% encapsulated. This differentiation was possible due to a confrontation with the F-MAD-*ON*-ZIF-8 quantification experiments, where all F-MAD can just be adsorbed and not encapsulated. The founded values are similar to the ones found in the literature for different encapsulated macromolecules, where usually no more than 50% of material is encapsulated [20,35]. The values are not significantly different from pH 5 and pH 7.4; however, the differences can be due to the different interactions and behaviors of the fluorophore at that pH.

The release kinetic profile of F-MAD was evaluated at pH 5 because this value closely mimics the natural acidic environment of the skin, which typically ranges from 4.5 to 5.5. This mildly acidic pH is crucial for maintaining the skin’s barrier function, inhibiting microbial growth, and promoting wound healing. Newly released papers [36] reported the importance of maintaining an acidic pH in the wound environment to facilitate healing. Therefore, the F-MAD@ZIF-8 sample was dissolved in citric acid–sodium citrate aqueous buffer (pH 5) under stirring, and at predetermined times, the F-MAD released into the buffer aliquots (a new aliquot was added to substitute the previous one at each time point) was quantified by spectroscopic signals.

**Figure 6:**
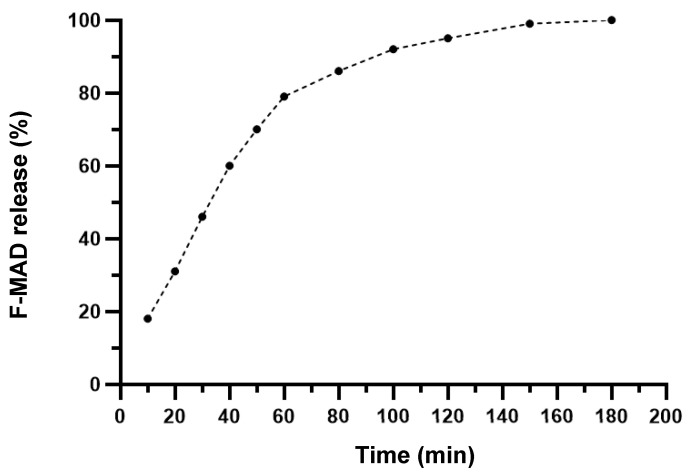
Release kinetic curve of F-MAD@ZIF-8 at pH 5 from t = 0 to t = 180 min. The experiments were repeated three times.

The release kinetics curve (Figure 6) indicates that approximately 18% of F-MAD is released within the first 10 min. Subsequently, between 10 and 60 min, an additional 10–15% release occurs. After the first hour, the release rate decreases, with about 10% of F-MAD being released every 20 min, culminating in complete release of the material at 180 min.

The data of this study have been analyzed, and two different kinetic models have been used to evaluate the best fitting (see Figure S10 for the model curves). Based on the R^2^ for the two models, R^2^ = 0.992 for Higuchi and R^2^ = 0.986 for the Korsmeyer–Peppas model, the results suggest that the Higuchi model has the highest degree of fit for our data.

### 3.4. Antibacterial Activity

**Figure 7:**
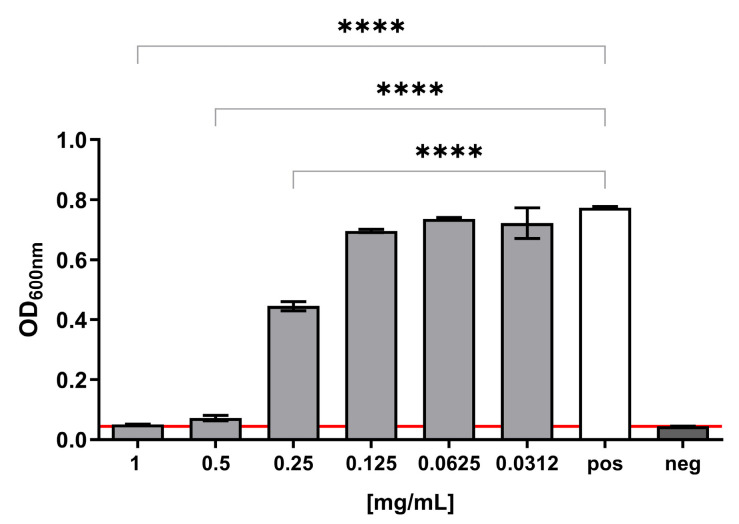
*S. epidermidis* growth in presence of different concentrations of MAD@ZIF-8. Positive control refers to bacterial growth in regular medium; negative control consists of sterile bacterial media. An arbitrary threshold was set at OD600nm = 0.045, corresponding to the mean value of the negative controls. Results are expressed as mean ± SEM of three independent experiments. **** *p* < 0.0001 significantly different from the positive control.

The antibacterial potential of MAD@ZIF-8 biocomposite was evaluated against *S. epidermidis* by determining the MIC value and assessing the bactericidal activity with a CFU count assay. *S. epidermidis* was chosen as model organism due to its previously demonstrated susceptibility to ZIF-8 crystals [4], making it a relevant system for evaluating the antimicrobial efficacy of the newly devised composite sample. The MAD@ZIF-8 biocomposite exhibited a strong antibacterial activity on *S. epidermidis*, with a MIC value of 1 mg/mL, and, at lower concentrations, a dose-dependent inhibitory effect on bacterial growth, with a statistically significant reduction at 0.5–0.25 mg/mL compared to the positive control (Figure 7).

**Figure 8:**
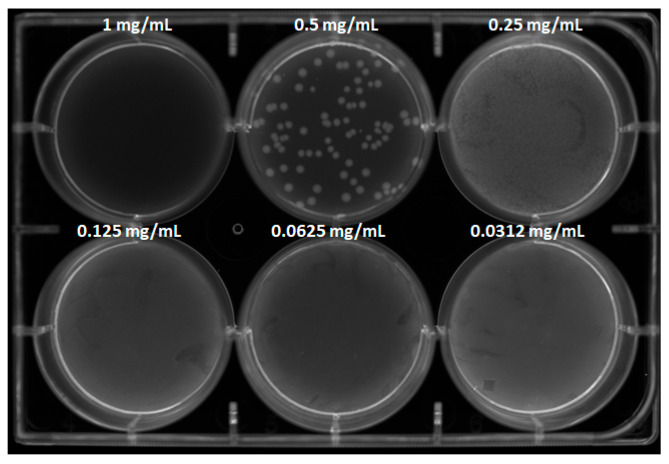
CFU count assay of *S. epidermidis* treated with MAD@ZIF-8 at different tested concentrations. Positive and negative controls and counting reports are available in Supplementary Materials S11 and S12.

Aliquots of the bacterial suspensions at different concentrations were spread on PCA plates, allowing viable cells to regrow (Figure 8). Colony count analysis revealed that MAD@ZIF-8 at 1 mg/mL exhibited bactericidal activity, as no colonies appeared on the plates; at 0.5 mg/mL, a 1 log_10_ reduction in bacterial count was observed compared to the positive control. These results were in agreement with those obtained in the liquid medium assay.

Given these findings, the performance of MAD@ZIF-8 at 1 mg/mL was compared with that of its individual components, ZIF-8 and MAD (Figure S13). Results obtained in liquid media showed reductions of 66.8 ± 0.7% and 59.7 ± 0.9%, respectively, indicating that the biocomposite exhibited enhanced antibacterial efficacy against *S. epidermidis*.

## 4. Conclusions

This study successfully demonstrated the encapsulation of snail slime from *Cornu aspersum* mucus (MAD) into Zeolitic Imidazolate Framework-8 (ZIF-8), using a biomimetic mineralization approach. This method, which mimics natural biomineralization processes, resulted in the formation of a MAD@ZIF-8 biocomposite. The research characterized this new composite and evaluated its potential as an antibacterial agent.

Fluorescence microscopy images showed that MAD was primarily located in the subsurface region of the ZIF-8 crystallites, rather than being uniformly distributed throughout the crystal.

The total immobilization efficiency was found to be approximately 98%. By comparing this with a sample where F-MAD was simply adsorbed onto pre-synthesized ZIF-8 (F-MAD-*ON*-ZIF-8), the study estimated that around 30% of the snail slime was effectively encapsulated within ZIF-8, while about 68% was adsorbed onto its surface. This encapsulation value is consistent with findings from similar studies involving other encapsulated macromolecules.

The release kinetic of the encapsulated material was evaluated by dissolving the composite in a citric acid–sodium citrate aqueous buffer at pH 5 [36]. The results showed a rapid initial release, with about 18% of F-MAD released within the first 10 min. This was followed by a slower, steady release, with 10–15% released in the first hour and then slowing down to 10% every 20 min. The complete release of F-MAD occurred after 180 min.

The antibacterial property of the MAD@ZIF-8 biocomposite was tested against *Staphylococcus epidermidis*, chosen as the model organism for this study as it had been previously used as a reference strain. MAD@ZIF-8 was bactericidal at 1 mg/mL and significantly reduced bacterial growth at 0.5 and 0.25 mg/mL. When the individual components of the composite material were assayed in liquid medium, they displayed a lower antibacterial potential against *S. epidermidis*, thus suggesting that encapsulating snail slime in ZIF-8 generates a markedly more effective antimicrobial agent in which the components act synergistically.

Further research could explore the precise mechanisms of the MAD-mediated aggregative growth and investigate the composite’s efficacy and mode of action against other bacterial strains and its potential in other biomedical fields, such as wound dressing and cancer therapy. A current limitation is the lack of comprehensive cytocompatibility data for the encapsulated material, which is crucial for determining its safety in biomedical applications. Future work will also primarily address this by performing cytotoxicity assessments on mammalian cell lines, such as osteoblasts. It is important to note that similar cytocompatibility studies were successfully conducted on the ZIF-8 material alone in our previous works [3,4]; therefore, a comparable rigorous evaluation is planned for this encapsulated composite to confirm its safe therapeutic window.

## Data Availability

The original data presented in the study are openly available in the Zenodo repository at https://doi.org/10.5281/zenodo.17618538, accessed on 1 November 2025.

## Supplementary Materials

The following supporting information can be downloaded at https://www.mdpi.com/article/10.3390/jfb16120443/s1, Figure S1: Fluorophore-tagged MAD aspect; Figure S2: ZIF-8 and Mad@ZIF-8 powders; Figure S3: Calibration curves of F-MAD; Figure S4: Thermogravimetric analysis (TGA) of as synthesized and activated samples; Figure S5: BET analysis of ZIF-8 and Mad@ZIF-8; Figure S6: Fluorophore-tagged sample ATR-FTIR; Figure S7: Fluorophore-tagged sample PXRD analysis; Figure S8: Fluorophore-tagged sample TGA; Figure S9: Fluorophore-tagged sample SEM; Figure S10: Kinetic models for the release study; Figure S11: CFU assay Analysis Report of MAD@ZIF-8 samples; Figure S12: CFU assay Analysis Report of control samples; Figure S13: *S. epidermidis* growth in different experimental conditions.

## Abbreviations

The following abbreviations are used in this manuscript:

MOF	Metal–Organic Framework
ZIF-8	Zeolitic Imidazolate Framework-8
MAD	“Madonita” snail slime
FITC	Fluorescein isothiocyanate
F-MAD	Fluorescein-tagged Madonita snail slime
2-HmIM	2-methylimidazole
Zn(mIM)_2_	Referred to Zeolitic Imidazolate Framework-8
PXRD	Powder X-Ray Diffraction
SEM	Scanning Electron Microscopy
ATR-FTIR	Attenuated Total Reflection–Fourier Transform Infrared
TGA	Thermogravimetric Analysis
MP-AES	Microwave Plasma Atomic Emission Spectrometry
BET	Brunauer–Emmett–Teller method
