# Supplementary material for: Encapsulation of Snail Slime in Metal–Organic Framework ZIF-8
Source: J Funct Biomater. 2025 Nov 28;16(12):443. doi: 10.3390/jfb16120443 (PMC12733450; doi:10.3390/jfb16120443)
Supplement: Supplementary file 1 [file jfb-16-00443-s001.zip › jfb-3967594-supplementary.pdf]

Supplementary Information for:

Encapsulation of Snail Slime in Metal Organic Framework ZIF-8

Maria Cristina Cassani [1,2], Francesca Bonvicini [3], Maria Francesca Di Filippo [4], Barbara Ballarin [1,2,5], Silvia Panzavolta [4,6,*] and Valentina Di Matteo [1,*]

[1] Department of Industrial Chemistry "Toso Montanari", University of Bologna, Via Piero Gobetti 85,
40129 Bologna, Italy; maria.cassani@unibo.it (M.C.C.); barbara.ballarin@unibo.it (B.B.)
[2] Center for Industrial Research-Advanced Applications in Mechanical Engineering and Materials Technology (CIRI-MAM), University of Bologna, Viale del Risorgimento 2, 40136 Bologna, Italy
[3] Department of Pharmacy and Biotechnology, University of Bologna, Via Massarenti 9, 40138 Bologna, Italy; francesca.bonvicini4@unibo.it
[4] Department of Chemistry "G. Ciamician", University of Bologna, Via Piero Gobetti 85, 40129 Bologna, Italy; maria.difilippo5@unibo.it
[5] Center for Industrial Research-Fonti Rinnovabili, Ambiente, Mare e Energia (CIRI-FRAME), University of Bologna, Viale del Risorgimento 2, 40136 Bologna, Italy
[6] Health Sciences and Technologies—Interdepartmental Center for Industrial Research (HST–ICIR), Alma Mater Studiorum—University of Bologna, Ozzano dell'Emilia, 40064 Bologna, Italy*
Correspondence: silvia.panzavolta@unibo.it (S.P.); valentina.dimatteo5@unibo.it (V.D.M.)

Table of Contents

S1. Fluorophore-tagged MAD aspect 1
S2. ZIF-8 and Mad@ZIF-8 powders 1
S3. Calibration curves of F-MAD 2
S4. Thermogravimetric analysis (TGA) of as synthesized and activated samples 3
S5. BET analysis of ZIF-8 and Mad@ZIF-8 4
S6. Fluorophore tagged sample ATR-FTIR 5
S7. Fluorophore tagged sample PXRD analysis 5
S8. Fluorophore tagged sample TGA 6
S9. Fluorophore tagged sample SEM 6
S10. Kinetic models for the release study 7
S11. iBright™ Imager Analysis Report of MAD@ZIF-8 samples........ 8
S12. iBright™ Imager Analysis Report of control samples........ 9
S13. *S. epidermidis* growths of MAD@ZIF-8, ZIF-8 and MAD samples........ 10

S1. Fluorophore-tagged MAD aspect

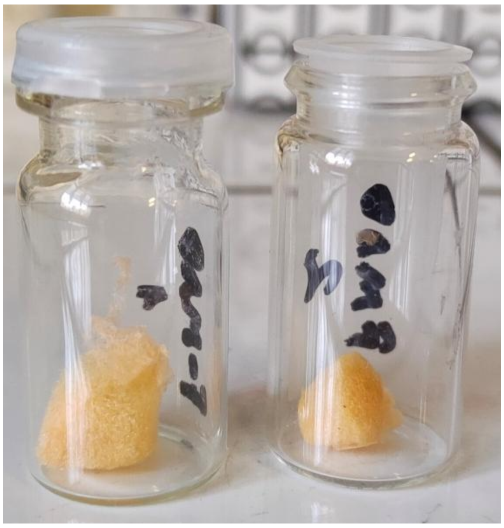

Figure S1. F-mad lyophilized samples

S2. ZIF-8 and Mad@ZIF-8 powders

As shown in *Figure S2.1*, the effective formation of the ZIF-8 can be seen at bare eye: the solution, from being transparent, becomes milky when the MOF is formed.

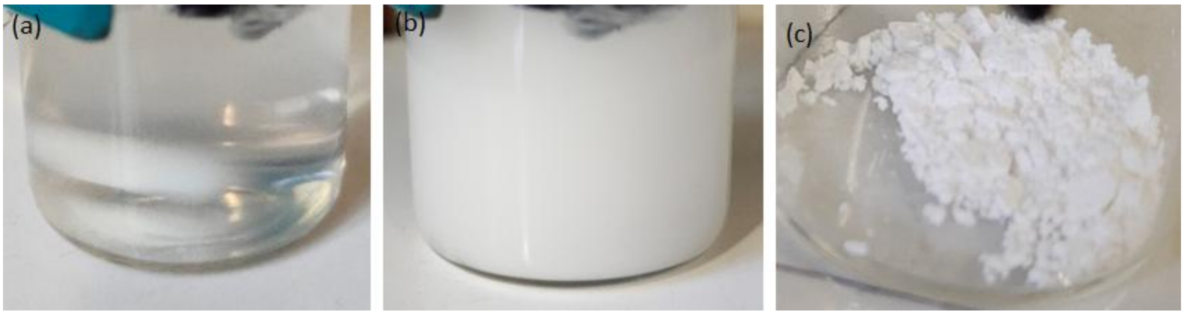

Figure S2.1. ZIF-8 starting solution (a), solution after 24h (b), final product powder (c)

Same is for the Mad@ZIF-8 sample (*Figure S2.2*) except for the fact that the presence of the protein makes the solution a bit coloured in a light beige tone.

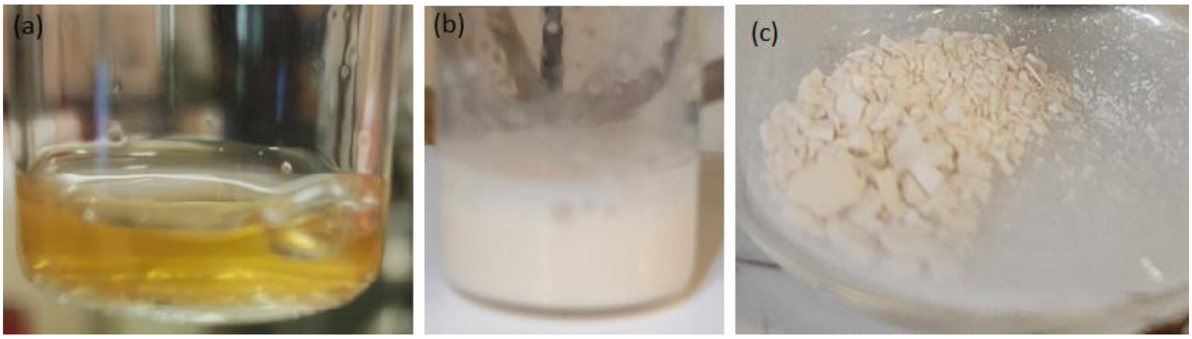

Figure S2.2. Mad@ZIF-8 starting solution (a), solution after 24h (b), final product powder (c)

S3. Calibration curves of F-MAD

The solvents were citrate buffer at pH 5 and PBS at pH 7.4. The citrate buffer (citric acid/sodium citrate) at pH 5 was prepared dissolving 7.26 g of sodium citrate tribasic dihydrate (MW 294.10 g/mol) in 240 mL of water and then adding to this solution citric acid monohydrate (MW 210 g/mol) measuring pH both with pH-metre and litmus paper.

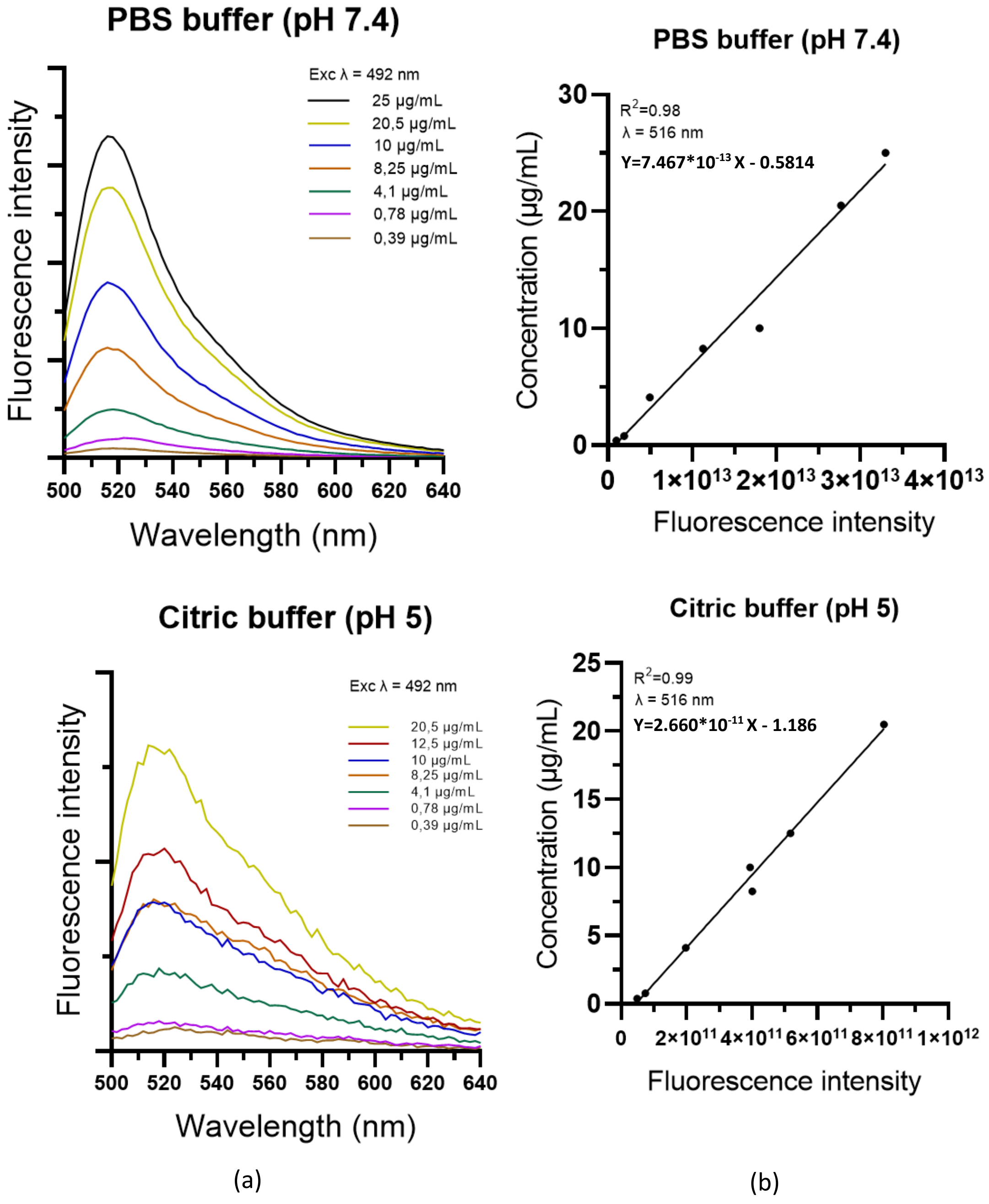

Figure S3. Fluorescence spectra of F-mad (a) and corresponding calibration curves (b) in PBS and Citric buffer

S4. Thermogravimetric analysis (TGA)

The thermogram of ZIF-8 and Mad@ZIF-8 as synthesized vs activated are shown in *Figure S4.1* and *S4.2. As-synthesized* ZIF-8

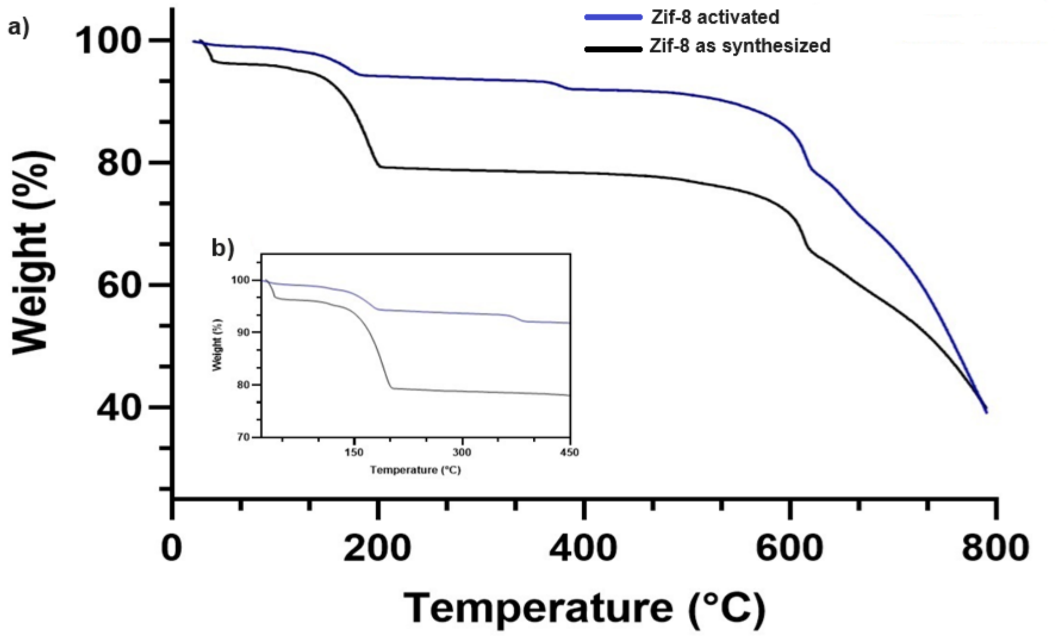

Figure S4.1 Overlay of the thermograms for the ZIF-8 as synthesized vs activated (a) and zoom of the region between 20 and 450°C (b)

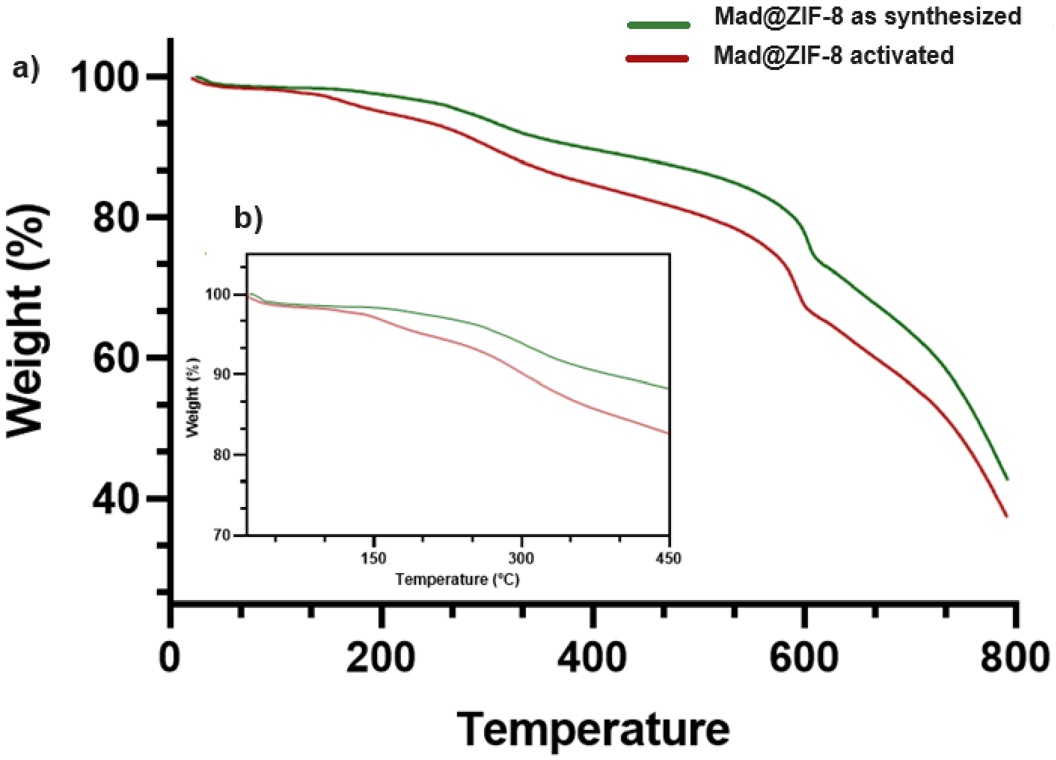

Figure S4.2 Overlay of the thermograms for the Mad@ZIF-8 as synthesized vs activated (a) and zoom of the region between 20 and 450°C (b)

Three significant weight losses are observed: the first corresponds to the evaporation of surface water, the second to the loss of structural water along with unreacted reagents, and the third aligns with the degradation of ZIF-8, which begins at 590°C. In the sample analyzed after BET, a reduction in the initial weight losses is noticeable, likely due to the sublimation of unreacted reagents during BET analysis that previously contributed to these early weight loss percentages.
Regarding the Mad@ZIF-8 sample, both visually and based on the temperature profile of the weight losses, it resembles the thermogram of the ZIF-8 post-BET. This suggests a reduced amount of structural water, possibly because macromolecules from the snail slime are encapsulated within the structure, and fewer unreacted reagents are present, as the slime occupies the pores.
It is also evident that the macromolecules encapsulated are protected by the ZIF-8 shell since the weight losses at 60°C and 200°C for the MAD (figure S4.3) are no longer as pronounced or noticeable in the Mad@ZIF-8 sample.

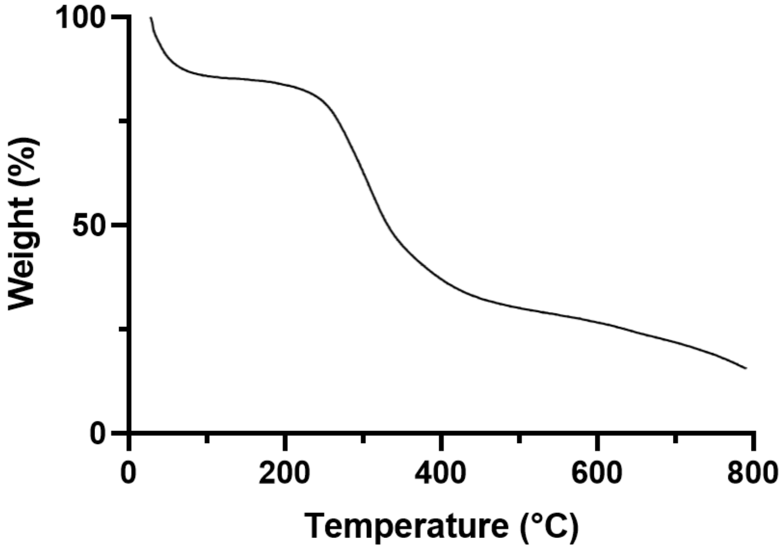

Figure S4.3 Thermogram for the MAD

S5. BET

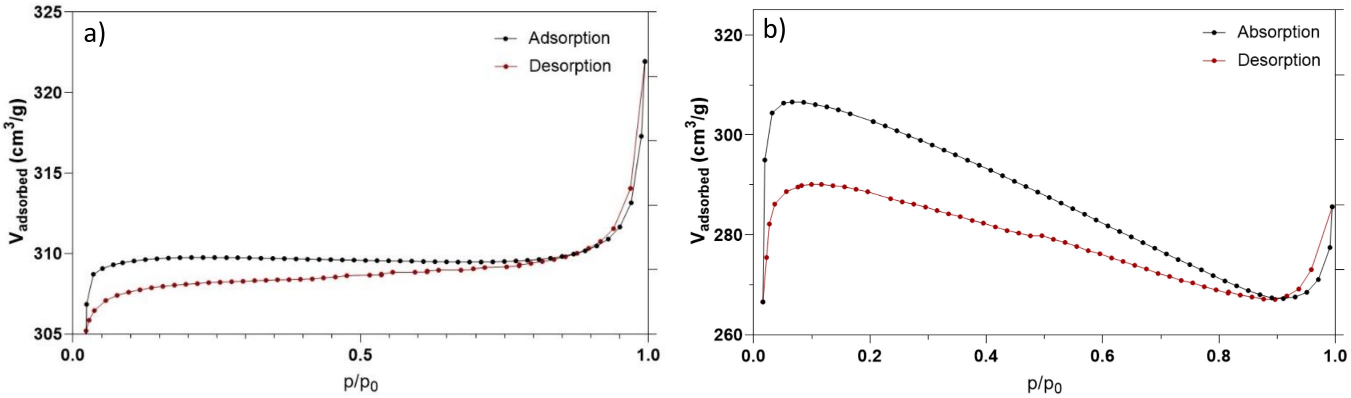

Figure S5. activated ZIF-8 (a) and activated mad@ZIF-8 (b) isotherms

S6. Fluorophore tagged samples ATR-FTIR

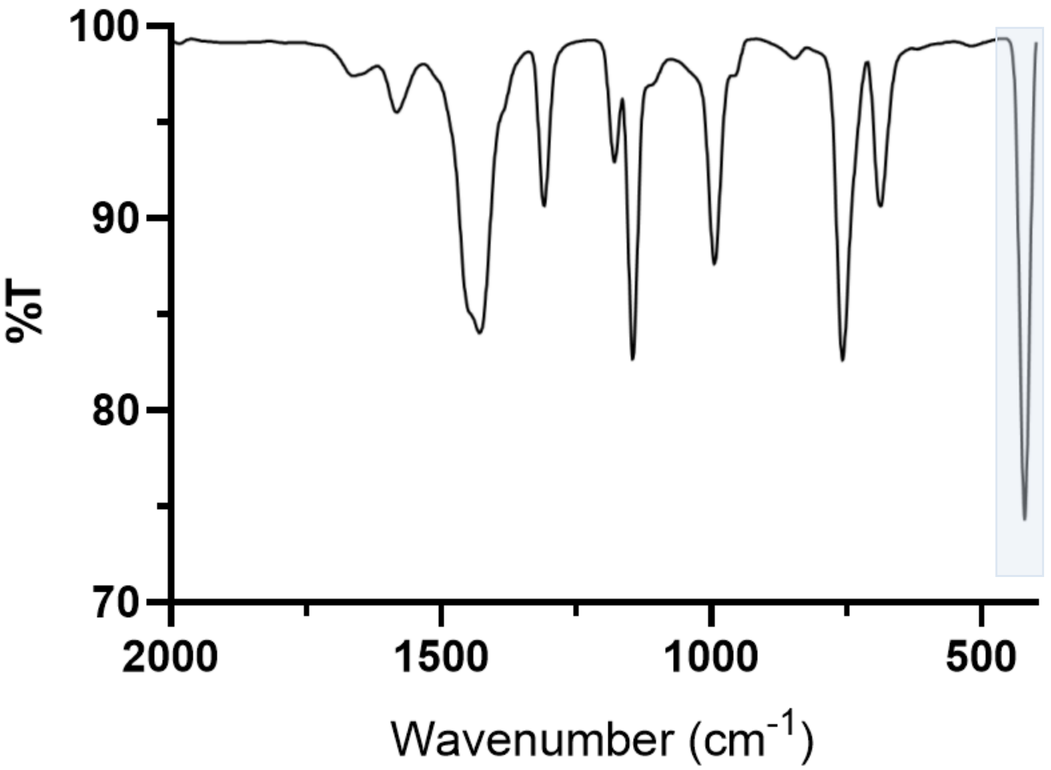

Figure S6. F-Mad@ZIF-8 FT-IR ATR spectrum (cut 2000-400 cm⁻¹)

S7. Fluorophore tagged samples PXRD analysis

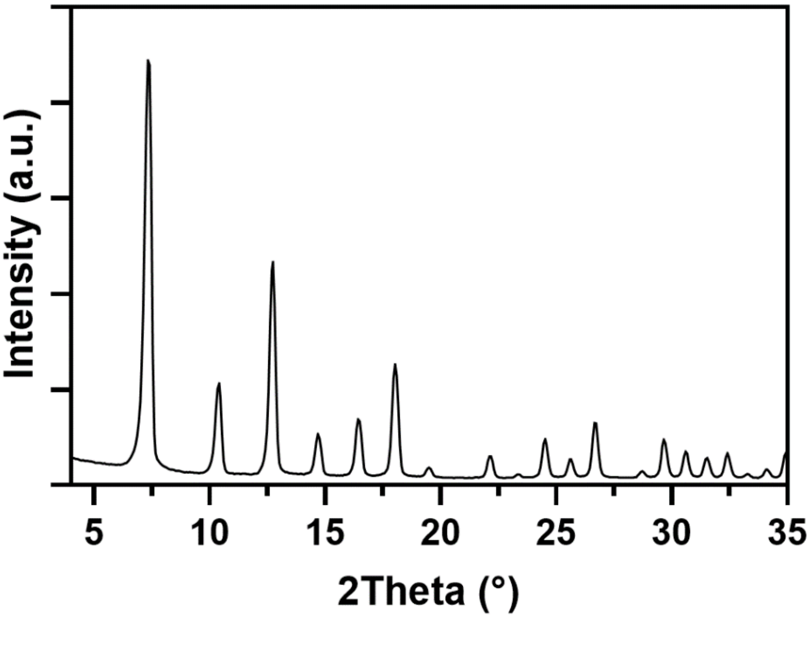

Figure S7. F-Mad@ZIF-8 diffractogram

S8. Fluorophore tagged samples TGA

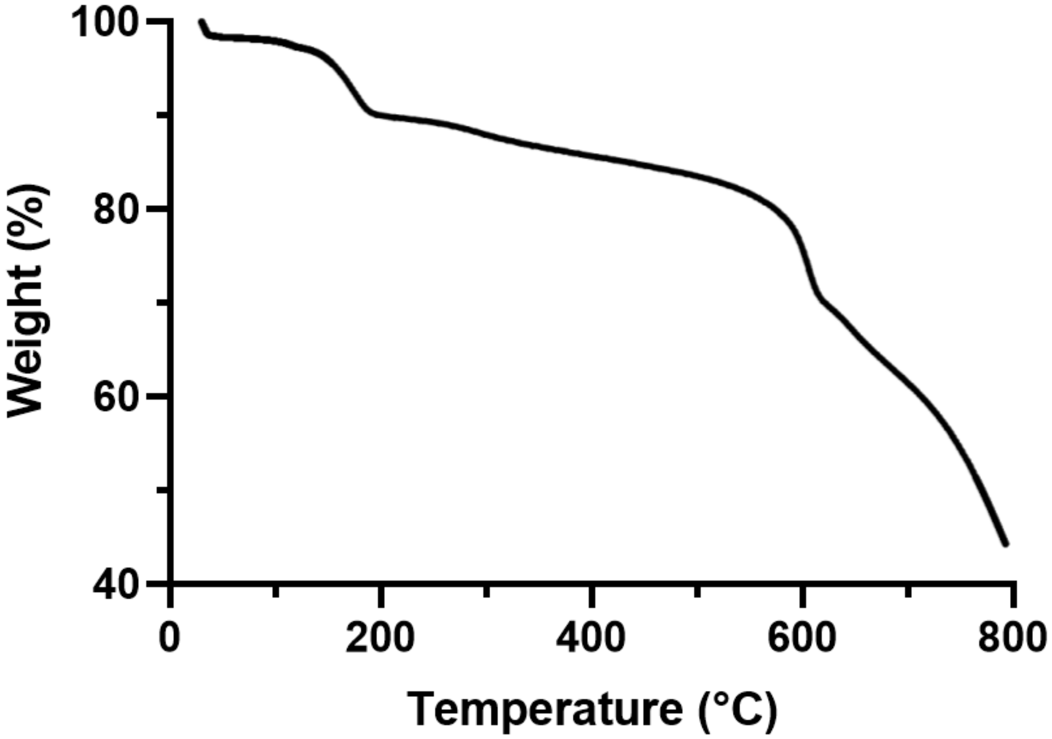

Figure S8. F-Mad@ZIF-8 thermogram

S9. Fluorophore tagged samples SEM

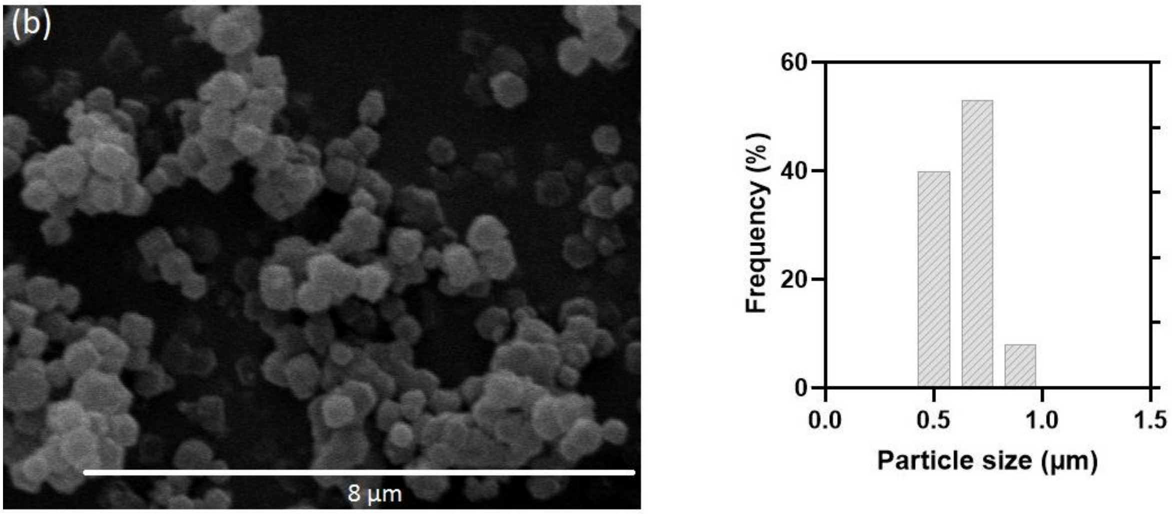

Figure S9. F-Mad@ZIF-8 SEM image and size distribution

S10. Kinetic models for the release study

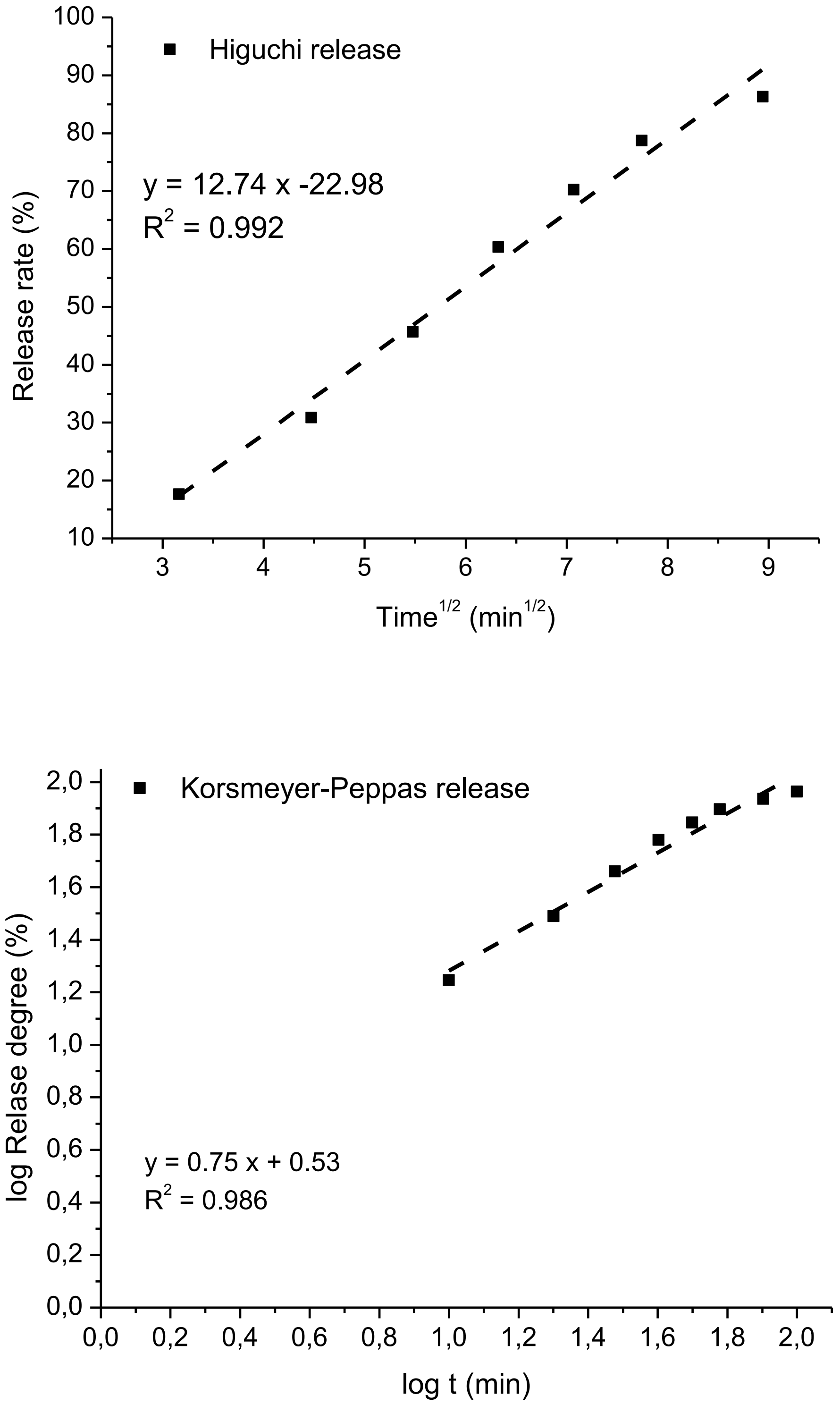

Figure S10. Higuchi and Korsmeyer-Peppas models for the release study.

S11. iBright™ Imager Analysis Report of MAD@ZIF-8 samples

W1 corresponds to MAD@ZIF-8 sample at 0.5 mg/mL

Date:	2025/08/01 10:58:14
Mode:	Universal
Notes:	No Comments
Model:	iBright™ FL1500
Inst Name:	2462624100001
Serial No:	2462624100001
Version:	1.8.2
User:	MicroBo
Exposure Time:	142 ms
Image area:	140.88mm x 112.70mm
Image size	1690px x 1352px
Optical Zoom:	1.6x
Digital Zoom:	1x
Focus level:	255
Dye:	Visible White colonies
Excitation:	490-520nm, Green Trans
Emission:	568-617nm
Resolution:	2 x 2

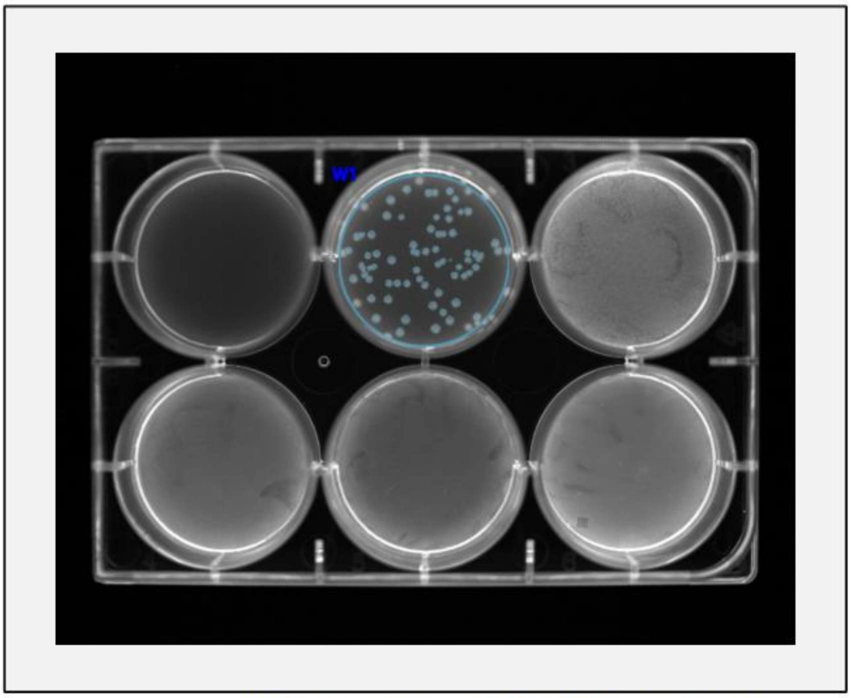

UNIVERSAL_08012025_105814_(Visible White colonies).tif

Colony Gating
Well 1
Size (Pixels): 7-281
Average Pixel Intensity: 16667-21252
Circularity: 0.4-1

UNIVERSAL_08012025_105814_(Visible White colonies).tif
COLONY COUNT ANALYSIS DATA TABLE

Channel 1 Number of Colonies (Channel): 70
Well 1 Number of Colonies (Well): 70

S12. iBright™ Imager Analysis Report of control samples

W1 corresponds to the positive control

Date: 2025/09/25 09:51:29
Mode: Universal
Notes: No Comments
Model: iBright™ FL1500
Inst Name: 2462624100001
Serial No: 2462624100001
Version: 1.8.2
User: MicroBo
Exposure Time: 146 ms
Image area: 112.70mm x 90.16mm
Image size 1690px x 1352px
Optical Zoom: 2x
Digital Zoom: 1x
Focus level: 355
Dye: Visible White colonies
Excitation: 490-520nm, Green Trans
Emission: 568-617nm
Resolution: 2 x 2

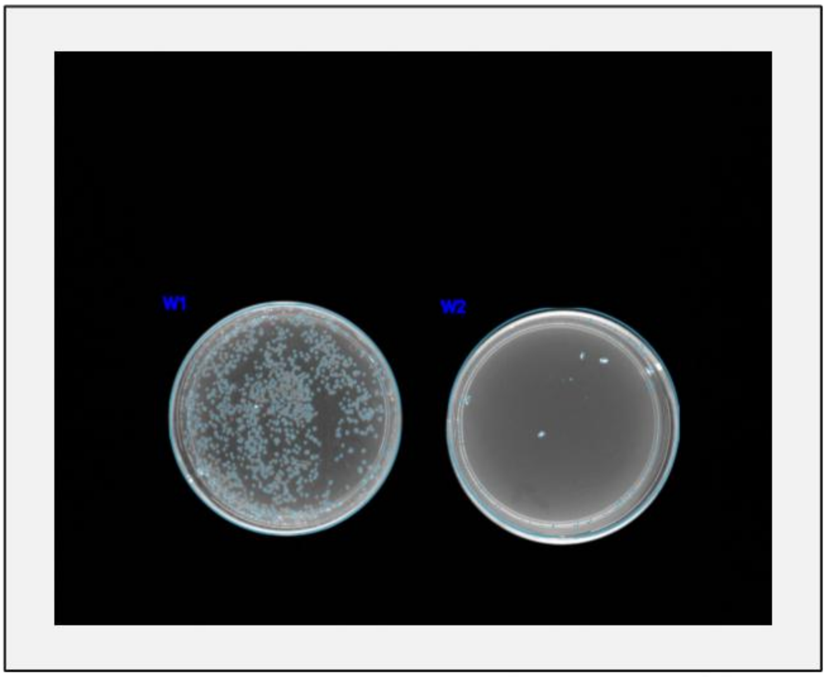

UNIVERSAL_09252025_095129_(Visible White colonies).tif

Colony Gating
Well 1
Size (Pixels): 5-169
Average Pixel Intensity: 18644-39701
Circularity: 0.25-1

Well 2
Size (Pixels): 5-155
Average Pixel Intensity: 16731-54470
Circularity: 0.25-1

UNIVERSAL_09252025_095129_(Visible White colonies).tif
COLONY COUNT ANALYSIS DATA TABLE

Channel 1 Number of Colonies (Channel): 929
Well 1 Number of Colonies (Well): 810

S13. *S. epidermidis* growths of MAD@ZIF-8, ZIF-8 and MAD samples

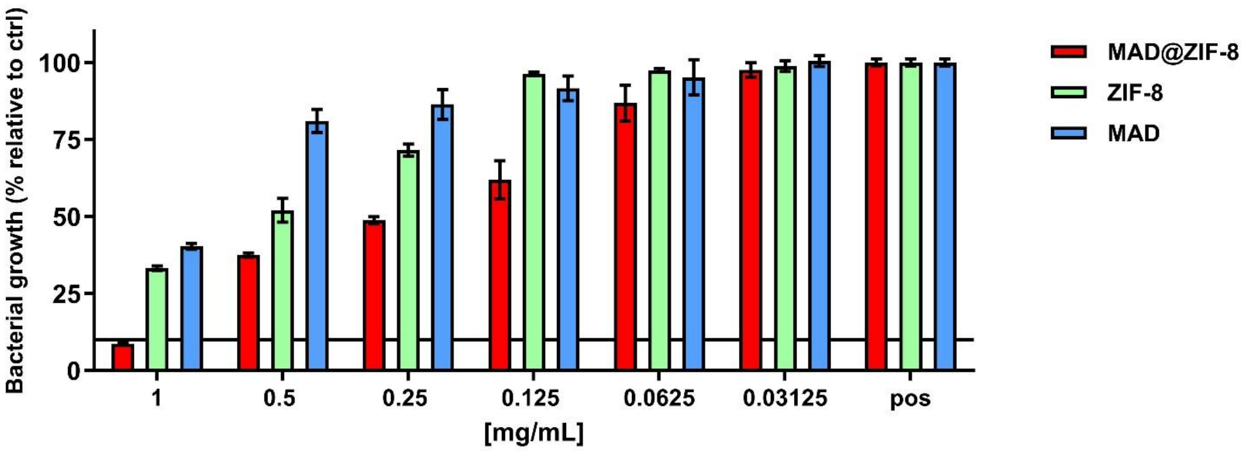

Figure S13. Bacterial growths (%) of S. epidermidis at the different experimental conditions. Positive control refers to bacterial growth in regular medium. An arbitrary threshold was set at 10% of growth. Results are expressed as mean ± SEM of three independent experiments.
